# Increased CD3^+^, CD8^+^, or FoxP3^+^ T Lymphocyte Infiltrations Are Associated with the Pathogenesis of Colorectal Cancer but Not with the Overall Survival of Patients

**DOI:** 10.3390/biology10080808

**Published:** 2021-08-20

**Authors:** Ana Margarida Barbosa, Olga Martinho, Rosete Nogueira, Juliana Campos, Liliana Lobo, Henrique Pinto, Adhemar Longatto-Filho, António G. Castro, Sandra F. Martins, Egídio Torrado

**Affiliations:** 1 Life and Health Sciences Research Institute (ICVS), School of Medicine, University of Minho, 4710-057 Braga, Portugal; id7167@alunos.uminho.pt (A.M.B.); olgamartinho@med.uminho.pt (O.M.); rosete.nogueira@med.uminho.pt (R.N.); farm.julianacampos@gmail.com (J.C.); lilianalobo1247@gmail.com (L.L.); henriquepinto325@gmail.com (H.P.); longatto@med.uminho.pt (A.L.-F.); acastro@med.uminho.pt (A.G.C.); sandramartins@med.uminho.pt (S.F.M.); 2 ICVS/3B’s—PT Government Associate Laboratory, 4710-057 Braga, Portugal; 3 CGC Genetics/Centro de Genética Clínica-Unilabs—Laboratory of Pathology, 4000-432 Porto, Portugal; 4 Laboratory of Medical Investigation (LIM) 14, Faculty of Medicine, University of São Paulo, São Paulo 01246-903, Brazil; 5 Molecular Oncology Research Center, Barretos Cancer Hospital, Barretos, São Paulo 14784-400, Brazil; 6 Coloproctology Unit, Braga Hospital, 4710-243 Braga, Portugal

**Keywords:** colorectal cancer, T lymphocytes, prognostic indicators, clinicopathological features, pathogenesis

## Abstract

**Simple Summary:**

Colorectal cancer (CRC) is amongst the deadliest cancers. Surgical excision of the primary tumor is the curative intent treatment; however, recurrence occurs in approximately 20% of patients. Therefore, novel staging protocols are crucial to inform clinicians which patients will recur. In this study, we explored the prognostic potential of tumor-infiltrating lymphocytes. Our data did not reveal any association between intratumor lymphocyte infiltrations with clinical or pathological data. On the other hand, the presence of CD3^+^, CD8^+^, or FoxP3^+^ lymphocyte infiltration in the tumor invasive margins were associated with markers of good prognosis. Despite this, we were not able to find any statistically significant alterations in the overall survival of patients, even though high infiltrations of FoxP3^+^ T lymphocytes in the tumor margin resulted in an increased overall survival of 14 months. Taken together, our data show that the location and type of tumor-infiltrating lymphocytes are associated with the pathogenesis of CRC; however, only high FoxP3^+^ T lymphocyte infiltrations are inclined to indicate favorable prognosis.

**Abstract:**

Tumor-infiltrating lymphocytes include heterogeneous populations of T lymphocytes that play crucial roles in the tumor immune response; importantly, their presence in the tumor tissue may predict clinical outcomes. Therefore, we herein studied the prognostic significance of the presence and location of CD3^+^, CD8^+^, and FoxP3^+^ T lymphocytes in colorectal cancer samples. In the intratumor analysis, our data did not reveal any association between lymphocyte infiltrations with clinical or pathological data. However, in the tumor margins, we found that the presence of high infiltrations of CD3^+^, CD8^+^, or FoxP3^+^ T lymphocytes were associated with TNM stages I-II (*p* = 0.021, *p* = 0.022, and *p* = 0.012, respectively) and absence of lymph node metastases (*p* = 0.010, *p* = 0.003, and *p* = 0.004, respectively). Despite these associations with good prognostic indicators, we were not able to find any statistically significant alterations in the overall survival of the patients, even though high infiltrations of FoxP3^+^ T lymphocytes in the tumor margins resulted in an increased overall survival of 14 months. Taken together, these data show that the presence of CD3^+^, CD8^+^, or FoxP3^+^T lymphocyte infiltrates in the tumor margins are associated with the pathogenesis of CRC, but only high Foxp3^+^ T lymphocyte infiltrations in the tumor invasive margins are inclined to indicate favorable prognosis.

## 1. Introduction

Colorectal cancer (CRC) is the third most common type of cancer worldwide, and its occurrence is responsible for nearly 10% of all deaths related to malignancies [1]. Historically, the incidence of CRC has been low at ages younger than 50 years; however, in recent years, there has been a rising incidence of CRC at these ages [2]. This emerging trend is prompting a rapid increase in the number of CRC cases in previously low-risk countries, a phenomenon ascribed to changes in dietary patterns and risk factors towards a more western lifestyle [3]. The cornerstone of curative intent treatment for CRC remains surgical excision of the primary tumor [4]. While this approach is curative for most patients, recurrence of CRC disease occurs in approximately 20% of patients [5]. Therefore, after tumor resection, clinicians often have follow-up appointments with patients to detect any recurrence at an early and treatable stage. These follow-ups have unraveled the need for novel predictive prognosis biomarkers and well-established staging protocols to inform clinicians which patients will recur.

The American Joint Committee on Cancer first defined the Tumor Node Metastasis (TNM) staging system to inform on patients’ prognosis [6]. Currently, TNM is the most widely used staging system for CRC, and its application in the clinic has been crucial to inform patients’ prognosis, also having a considerable and direct impact on the treatment that patients receive [6]. However, clinical evidence suggests that the outcome of the disease varies significantly among patients within the same TNM stage [7,8,9]. This is particularly noticeable at TNM stage II, wherein one-third of all patients may still die of recurrent disease [10,11]. Contrarily, patients at TNM stage III may be cured of the disease by surgery alone [12]. As such, under or overtreatment may occur between stages determined by the TNM system, as previously demonstrated [13,14]. Additionally, the lack of consensus on the application of TNM staging and the constant update of revised versions are significant hurdles in comparing different cohorts [9,15,16]. Therefore, clinical practice may benefit from the inclusion of other staging methodologies to discriminate patients who may benefit from additional therapies, such as adjuvant chemotherapies.

Over the last decade, there has been a progressive increase in our understanding of the tumor microenvironment, which prompted the identification of key players of the immune response to tumors. Particularly important and with prognostic potential in CRC are tumor-infiltrating lymphocytes (TIL), which are heterogeneous populations of T lymphocytes present in the tumor microenvironment [17,18,19,20,21]. In this regard, the presence of CD8^+^ T lymphocytes has been associated with good prognosis in different types of solid tumors [22,23]. This T lymphocyte population mediates anti-tumor activity through antigen-specific cytotoxicity and by producing anti-tumor cytokines, namely IFN-γ and TNF-α [24,25]. On the other hand, increased tumor infiltration by FoxP3-expressing T lymphocytes has been associated with reduced overall survival of patients with different types of cancer, including breast [26], lung [27], and cervical cancers [28]. However, this association is not seen in all cancer types, as FoxP3^+^ T lymphocyte infiltrates have been associated with good prognosis in other cancers, such as head and neck cancers [29]. In colorectal cancer, FoxP3^+^ T lymphocyte infiltrates have been associated with good and bad prognosis by different studies [30,31,32,33]. Taken together, these data warrant further investigation on the prognostic potential of FoxP3^+^ T lymphocytes in colorectal cancer.

The analysis of immune infiltrates and their correlation with patients’ pathological records originated the development of staging methods based on the intratumor and invasive margin infiltration of CD3^+^ and CD8^+^ T lymphocytes [34]. While the predictive capacity of TNM staging is more reliable than alternative methods, such as DNA content or genetic features, the analysis of tumor immune infiltrate has been suggested to surpass the TNM classification in multivariate analyses [35]. Indeed, after adjusting for TNM stage, recent data suggest that the density of CD3^+^ T lymphocytes remained as an independent prognostic factor [36]. Furthermore, patients with low numbers of tumor-infiltrating CD8^+^ T lymphocytes relapsed more independently of the T stage of the tumor [19]. These data demonstrate the high prognostic utility of TILs in staging CRC patients. However, as discussed above, the interaction between different populations of TILs in the tumor or invasive margin may influence tumor progression or control. As such, it is crucial to define the prognostic utility of the different populations of T lymphocytes.

In this work, we analyzed the presence of lymphocyte infiltrates, specifically CD3^+^, CD8^+^, and FoxP3^+^ T lymphocytes, in CRC tumors including their invasive margins to evaluate their association with clinicopathological information and overall survival of patients. We did not find any associations between the presence and extent of intratumor T lymphocyte infiltrations with the clinical or pathological data of the patients. On the other hand, the infiltration of CD3^+^, CD8^+^, or FoxP3^+^ lymphocytes in the tumor invasive margins were associated with the pathogenesis of CRC, but only FoxP3^+^ T lymphocyte infiltrations were inclined to indicate favorable prognosis.

## 2. Materials and Methods

### 2.1. Patients Specimens

A total of 194 samples of colorectal cancer (CRC) at stage I to IV were used in this retrospective study. These samples were collected from patients diagnosed with CRC that underwent surgical excision of the primary tumor at the Hospital of Braga, Portugal, between January 2005 and January 2010. The CRC tissue extracted during the surgery was formalin-fixed and embedded in paraffin. Clinical and pathological data was available for 184 cases and was obtained through medical charts and pathology reports (Table 1).

### 2.2. Immunohistochemistry

Tumor-infiltrating lymphocytes were detected by immunohistochemistry using antibodies against CD3 (MCA1477, BioRad), CD8 (ab4055, Abcam), or FoxP3 (14477782, Invitrogen). Briefly, whole-tissue sections were deparaffinized and hydrated to prepare the tissue for the staining. The slides from whole-tissue sections were incubated for 30 min in citrate buffer at 96 °C followed by incubation in hydrogen peroxide for 10 min at room temperature (RT). The slides were then incubated for 1 h with blocking solution (PBS with 5% BSA and 0.05% Tween 20) before incubation with primary antibodies at 4 °C for 16 h. After washing with PBS, the slides were incubated with biotinylated-secondary antibodies for 1 h at RT. Slides were then incubated with streptavidin for 1 h followed by an incubation with chromogen (DAB; Dako) for 10 min, and then counterstained with hematoxylin. Amygdala sections were used as positive controls.

Immunostaining analysis was used to determine the presence of CD3, CD8, or FoxP3 infiltrates in the tumor, tumor invasive margins, and normal adjacent colon epithelium using an Olympus BX41 microscope. Immunostaining was considered positive whenever there was cytoplasmatic and membrane staining for CD3, membrane staining for CD8, and nuclei staining for FoxP3. Grading of the immunostaining was performed in a blind fashion by consensus of two experienced pathologists, without having prior knowledge of the pathological stage or any other clinical or follow-up data for each case. Briefly, all samples were first analyzed under the microscope at a magnification of 100× to determine the extent of infiltration for each marker. From this analysis, the grade of CD3 and CD8 infiltration was categorized as <10%, 10–50%, or >50%, and the grade of FoxP3 infiltration was categorized as <10%, 10–30%, or >30%, as the expression of this marker was less extensive than of CD3 or CD8. Ten high magnification fields (×400) from each region of the tumor were then semi-quantitatively analyzed to determine the number of lymphocytes that stained positive for each marker.

**Figure 1:**
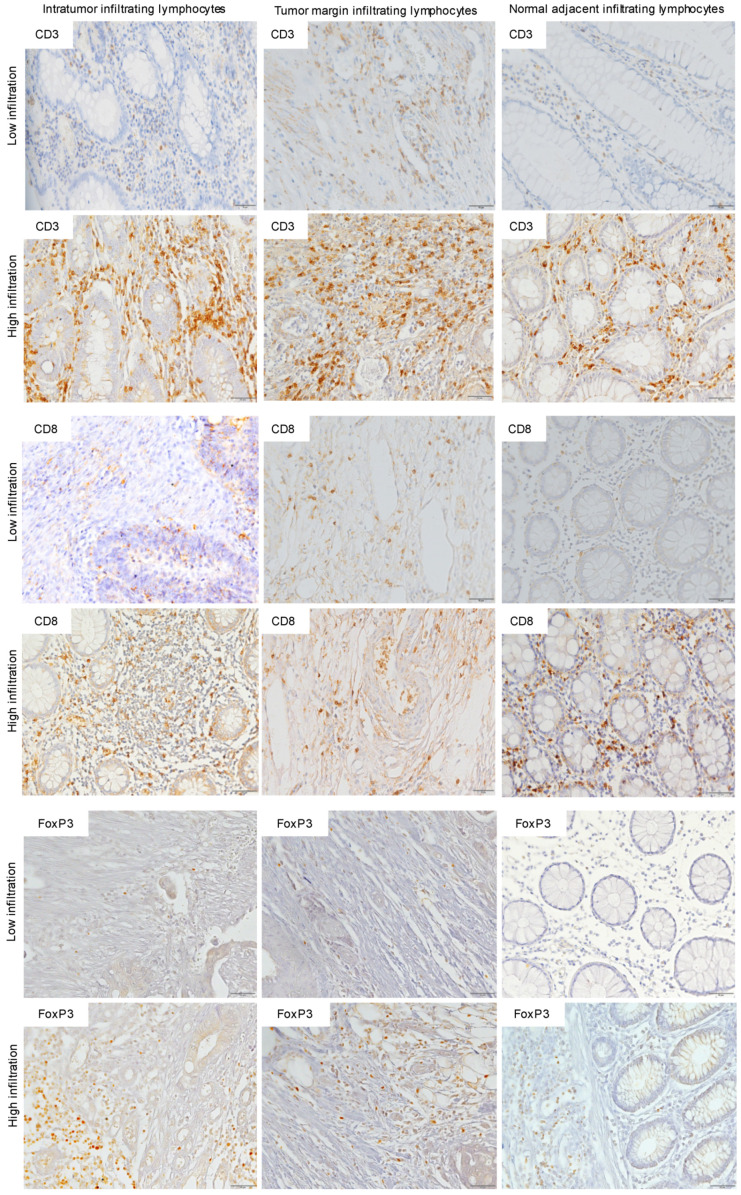
Immunohistochemistry (IHC) of CD3, CD8, and FoxP3 T lymphocytes in CRC. Representative IHC showing low and high CD3^+^, CD8^+^, and FoxP3^+^ T lymphocytes densities. Magnification: 200×.

For statistical analysis, each section was then classified as either low (<50%) or high (>50%). Representative images (Figure 1) were obtained under brightfield microscopy (Olympus BX61) and were recorded with a digital camera (DP70) using the Cell∧P software.

### 2.3. Statistical Analysis

Statistical analysis was performed using the Statistical Package for the Social program Science (SPSS), version 24.0, SPSS Inc.^®^, Chicago, IL, USA.

Simple descriptive analyses were performed, determining the total number of cases and relative frequencies for each clinical-pathological factor. To assess the existence of any association of clinical or pathological data with immunohistochemistry results, Pearson’s chi-square test and Fisher’s exact test (scattered data) were performed. Survival analysis was performed using Kaplan-Meier curves and significant differences were determined by the log-rank test. Survival was defined between the period of analysis and death from any cause. Patients who quit the study were censored on the date of the last contact. Confidence values (*p*) below 0.05 were considered statistically significant.

### 2.4. Ethics Statement

The study was approved by the Ethics Committee for Research in Life and Health Sciences at University of Minho (CEICVS 004/2020) and by the Ethics Committee of Hospital de Braga (32/2013).

## 3. Results

### 3.1. CD3^+^, CD8^+^, or FoxP3^+^ T Lymphocyte Infiltrations Are Higher in the Tumor Tissue Than in the Normal Adjacent Tissue

To explore the association between tumor-infiltrating T lymphocyte populations with CRC prognosis, we determined the infiltration of CD3^+^, CD8^+^, or FoxP3^+^ T lymphocytes within the tumor, tumor margins, and in the normal adjacent tissue by immunohistochemistry. We began by analyzing the extension of immunostaining for the different lymphocyte markers in the different regions of the samples (Table 2). This analysis revealed a high intratumor infiltration of CD3^+^ T lymphocytes in 53% (103/194) of samples. When we compared the proportion of infiltrating lymphocytes that were CD8^+^ or FoxP3^+^, we found a high intratumor infiltration of CD8^+^ T lymphocytes in 52% (97/187) and a high intratumor infiltration of FoxP3^+^ T lymphocytes in 53% (103/196) of samples (Table 2). Regarding the infiltration of lymphocytes in the tumor margin, we also found a high infiltration of CD3^+^ T lymphocytes in 59% (113/192), CD8^+^ T lymphocytes in 58% (107/185), and FoxP3^+^ T lymphocytes in 59% (114/194) of samples (Table 2). On the other hand, the extension of T lymphocyte infiltration in the normal adjacent tissue was lower than in the tumor tissue. Indeed, we found a high infiltration of CD3^+^ T lymphocytes only in 43% (61/143), CD8^+^ T lymphocytes in 41% (57/139), and FoxP3^+^ T lymphocytes in 42% (62/146) of samples (Table 2).

We then determined the association between lymphocyte infiltration within the different tissues analyzed. As shown in Table 3, the high intratumor infiltration of CD3^+^ T lymphocytes correlated with high infiltration of the same population in the tumor margin (*p* < 0.001). Similarly, the high infiltration CD3^+^ T lymphocytes of the tumor margin correlated with high infiltration of the same population in the normal adjacent tissue (*p* < 0.001). However, there was no correlation between the intratumor infiltrations of CD3^+^ T lymphocytes with the infiltration of these cells in normal adjacent tissue, indicating that these cells were actively recruited to the tumor. The same results were obtained for CD8^+^ and FoxP3^+^ T lymphocytes (*p* < 0.001 for all samples).

Taken together, these data show that a significant proportion of samples had high T lymphocyte infiltrates and that the profile of infiltration is similar between the tumor and the margins. Furthermore, the infiltration of lymphocytes increased from the normal adjacent tissue to the tumor tissue.

### 3.2. High Intratumor Infiltration of FoxP3^+^ T Lymphocytes in Less Severe CRC Lesions

With the above data showing the presence of TIL in all tumor samples analyzed, we then sought to determine whether the degree of intratumor lymphocyte infiltration influenced CRC clinical outcome. To do this, we correlated the extension of intratumor infiltration of CD3^+^, CD8^+^, or FoxP3^+^ T lymphocytes with the clinicopathological information of CRC patients.

Our analysis revealed no association between the clinical parameters with the extension of intratumor lymphocyte infiltration for all the immune populations analyzed (Table 4).

We then analyzed the association between the pathological parameters with the extension of intratumor lymphocyte infiltrations (Table 5). As for clinical parameters, our analysis did not find any association between pathological parameters and the extension of intratumor T lymphocyte infiltration for all the immune populations analyzed (Table 5).

These data show that the extent of intratumor T lymphocyte infiltrations does not impact the pathogenesis of CRC.

### 3.3. High CD3^+^, CD8^+^, or FoxP3^+^ T Lymphocytes in the Tumor Margins Are Associated with Good Prognostic Indicators

As we found high lymphocyte infiltrates in the tumor margins of a significant number of samples, we next determined if the accumulation of CD3^+^, CD8^+^ or FoxP3^+^ T lymphocytes were associated with any CRC clinical or pathological parameters.

Our analysis did not uncover any association with clinical parameters (Table 6).

However, when we did the same analysis for pathologic parameters, we found that high accumulation of CD3^+^ T lymphocytes was associated with normal levels of carcinoembryonic antigen (CEA) (*p* = 0.026), an important biomarker of different types of cancer, including CRC [37] (Table 7). High infiltration of FoxP3^+^ or CD8^+^ T lymphocytes also showed a tendency with normal CEA levels (*p* = 0.057 and *p* = 0.053, respectively), with *p*-values very close to the statistical threshold. Additionally, the absence of lymph node metastasis was positively correlated with a high accumulation of CD3^+^, CD8^+^, or FoxP3^+^ T lymphocytes (*p* = 0.01, *p* = 0.004, and *p* = 0.003, respectively). We also found a tendency associating the presence of high CD3^+^ infiltration with the absence of distant metastases (*p* = 0.054). Finally, the high infiltration of CD3^+^, CD8^+^, and FoxP3^+^ T lymphocytes was associated with TNM stages I-II (*p* = 0.021, *p* = 0.022, and *p* = 0.012, respectively). Together, these results show that high infiltrations of CD3^+^, CD8^+^, or FoxP3^+^T lymphocytes in the tumor margins are associated with good prognostic indicators.

### 3.4. The Presence of High CD3^+^, CD8^+^, or FoxP3^+^ T Lymphocyte Infiltrations Inside the Tumor or in Its Invasive Margins Does Not Impact the Overall Survival of Patients

To determine whether lymphocyte infiltrations impacted the patients’ overall survival, we performed log-rank tests and constructed Kaplan–Meier curves.

**Figure 2:**
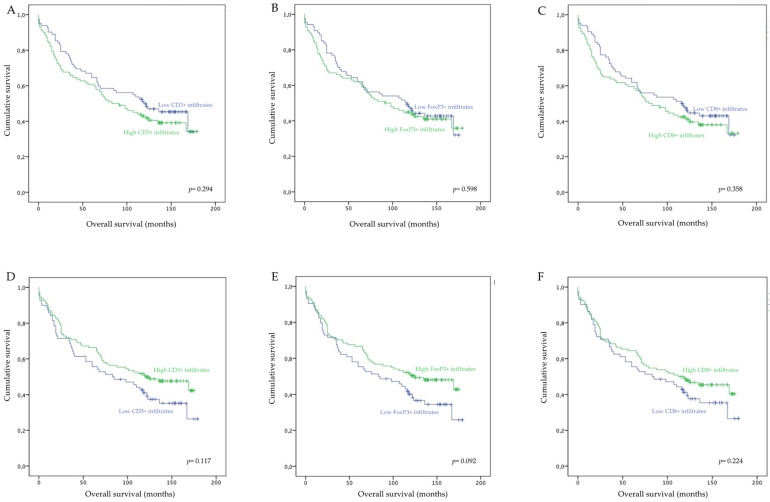
Overall survival of CRC patients according to low or high densities of intratumor and tumor margin infiltrating CD3^+^, FoxP3^+^, and CD8^+^ T lymphocytes. (**A**) Overall survival according to intratumor CD3^+^ T cell density; (**B**) Overall survival according to intratumor FoxP3^+^ T cell density; (**C**) Overall survival according to intratumor CD8^+^ T cell density; (**D**) Overall survival according to tumor margin CD3^+^ T cell density; (**E**) Overall survival according to tumor margin FoxP3^+^ T cell density; (**F**) Overall survival according to intratumor CD8^+^ T cell density.

Our analysis did not reveal any statistically significant association between the extension of intratumor (Figure 2A–C) or tumor margin (Figure 2D–F) lymphocyte infiltrations with the overall survival of patients. These data indicate that, in our cohort, tumor-infiltrating lymphocytes do not impact the clinical outcome of CRC patients. Interestingly however, while not statistically significant, our analysis revealed that high infiltrations of CD3^+^, CD8^+^, or FoxP3^+^ T lymphocytes in the tumor margins resulted in increased overall survival of 13.4 (*p* = 0.117), 7 (*p* = 0.224), and 14 months (*p* = 0.092), respectively. On the other hand, high intratumor infiltrations of CD3^+^, CD8^+^, or FoxP3^+^ resulted in a slight decrease in patients’ overall survival of 13.5 (*p* = 0.294), 9.3 (*p* = 0.358), and 5.3 months (*p* = 0.598), respectively.

These data show that, while the intratumor infiltration by FoxP3^+^ lymphocytes and infiltration of the tumor margins by CD3^+^, CD8^+^, or FoxP3^+^ T lymphocytes are associated with good prognostic indicators, they do not impact the overall survival of patients.

## 4. Discussion

The interaction between tumor cells and the immune system has prompted the quantification of immune infiltrates, particularly T lymphocytes, as prognostic markers for colorectal cancer (CRC) [35]. Herein, we analyzed the presence of lymphocyte infiltrates (CD3^+^, CD8^+^, or FoxP3^+^ T lymphocytes) inside the tumor and the tumor invasive margins of CRC samples to evaluate their prognostic potential. We were not able to find any associations between the presence and extent of intratumor T lymphocyte infiltrates with the clinical or pathological data of the patients, indicating that, at least in our cohort, intratumor lymphocytes do not influence the pathogenesis of CRC. Crucially, when we perform the same analyses for the tumor margins, we found that the presence of high CD3^+^, CD8^+^, or FoxP3^+^ T lymphocyte infiltrates were associated with TNM stages I-II, non-invasion of lymph nodes, and normal CEA levels. These data suggest that the presence of CD3^+^, CD8^+^, or FoxP3^+^ T lymphocytes in the tumor invasive margins are associated with good prognostic indicators; however, we could not demonstrate any significant association between any of the T lymphocyte population analyzed and the overall survival of the patients.

In recent years, the analysis of the immune reaction inside the tumor and its invasive margins has been suggested to predict disease-free survival and overall survival of CRC patients, independently of the local extent of the tumor or the invasion of regional lymph nodes (TNM stages I, II, and III) [36]. The prognostic potential of the tumor immune reaction prompted the development of methodologies to quantify, in situ, the extent of immune infiltrates, particularly CD3^+^ and CD8^+^ T lymphocytes [35]. Several studies show that high infiltrations of CD3^+^ and/or CD8^+^ T lymphocytes within CRC tumors and their invasive margins were associated with early stages of the disease (TNM stages I-II) and other good prognostic indicators, including absence of lymph node metastasis and distant metastasis [19,38]. Our study corroborated these observations but only for CD3^+^ or CD8^+^ T lymphocyte infiltrates in the tumor margins. Indeed, we were unable to find any association between the presence of CD3^+^ or CD8^+^ T lymphocyte infiltrates inside the tumor with markers of good CRC prognosis. In this regard, previous data has shown that, while CD8^+^ T lymphocytes are directly capable of killing tumor cells and positively affect prognosis in a broad range of tumors [39,40,41,42], several other studies have shown no such correlation with prognosis [43,44]. As such, it is possible that the tumor microenvironment could modulate the effector function of CD8^+^ T lymphocytes, and that this effect may depend on environmental variables such as the microbiome [45,46] or the tumor inflammatory status [47]. In this regard, alteration in the intestinal microbiota has been shown to increase intestinal tumorigenesis by enhancing inflammation and promoting T cell exhaustion [48]. Therefore, in addition to determining the presence of CD8^+^ T lymphocytes, future studies should also focus on determining their effector function.

As discussed above, we also found that the high accumulation of FoxP3^+^ T lymphocytes in the tumor margins was associated with TNM stages I-II, normal CEA levels, and, more importantly, with the non-invasion of lymph nodes. While FoxP3 can be transiently expressed by recently activated T cells in humans, the expression of this transcription factor also marks a population of regulatory T lymphocytes that can downregulate immune responses and, consequently, dampen anti-tumor immune mechanisms [49,50,51]. Since we did not evaluate the function of FoxP3^+^ lymphocytes, we were not able to discriminate between the regulatory and non-regulatory populations of FoxP3^+^ lymphocytes. This is an important limitation of our study, as it prevented us from drawing any conclusion on the prognostic potential of the regulatory population of FoxP3-expressing lymphocytes. However, we suggest that the immunosuppressive capacity of the regulatory population of FoxP3-expressing lymphocytes may be important in CRC to control continuous and aggressive inflammatory responses that may favor tumor proliferation [52,53]. Our data are in accordance with previous data showing that high frequencies of FoxP3^+^ T lymphocytes are associated with early T stages and absence of lymph node involvement [31]. However, other studies also found associations between high FoxP3^+^ T lymphocyte infiltrations and increased survival of CRC patients [32,54], which we did not find. Despite this, the high accumulation of these cells in the tumor margins resulted in an average gain of 14 months of the patients’ life expectancy (*p* = 0.092). It is important to note that previous studies have also reported an association between high FoxP3^+^ lymphocyte infiltrations and advanced CRC [33,55]. Additionally, the presence of FoxP3^+^ T lymphocyte infiltrates were associated with poor prognosis in different types of cancers, including breast [26], lung [27], pancreatic [56], ovarian [57], and cervical [28] cancers. These data show that the presence of FoxP3^+^ T lymphocytes is not always associated with a good prognosis. As this population may downregulate immune responses, we suggest that their protective effect and their prognostic potential may depend on the inflammatory status of the tumor. As such, future research is required to consolidate the prognostic significance and the context wherein FoxP3^+^ T lymphocytes have prognostic significance.

Our data point to the location of immune cells in relation to the tumor as an important factor for prognosis. Indeed, while we did not find any association between intratumor T lymphocytes with clinical or pathological data in the tumor margins, the extent of CD3^+^, CD8^+^, and FoxP3^+^ lymphocyte infiltrations were all associated with good prognostic indicators. It is tempting to speculate that the intratumor-infiltrating T lymphocytes may be modulated by the tumor microenvironment, while in the tumor margins, these populations are able to maintain their functions for longer periods. As discussed above, this may explain the lack of association between the high intratumor infiltration of CD8^+^ T lymphocytes and markers of good prognostic or even overall survival seen in other studies [32,35,38]. Taken together, the distribution of immune cells, as well as their functional capability, may be important in predicting patients’ prognosis.

The potential limitations in analyzing tumor-infiltrating lymphocytes and perform direct comparisons with other studies include the various criteria used in different studies and the heterogeneity in the patterns of tumor-infiltrating lymphocytes [58]. In our study, we accounted for this heterogeneity by staining large samples of tumor tissue with the different lymphocyte markers, including samples with normal adjacent tissue. The assessment of the immunostainings and analysis of the extent of lymphocyte infiltration in the different areas of the tumor by an experienced pathologist ensured that the heterogeneous expression patterns of the different markers used were seen in the context of the entire tumor section. Moreover, automated counting methods, which are not available in all laboratories and have been used in multiple studies, may not yield results comparable to the usual method. In all, further studies need to take into account the heterogeneity of lymphocyte infiltrates and evaluating methods.

In conclusion, our results indicate that only the presence of high infiltrates of CD3^+^, CD8^+^, and FoxP3^+^ T lymphocytes in the tumor invasive margins are associated with good prognostic indicators and potentially limit the aggressiveness and spread of CRC. The presence of lymphocyte infiltrates inside the tumor was not associated with any clinical or pathological parameter. However, while previous studies showed an association between high lymphocytic infiltrations, particularly CD8^+^, and survival of CRC patients [35,38,59], we were unable to find any association between intratumor or tumor invasive margin lymphocyte infiltrations and overall survival. As such, while the consensus is that the tumor immune reaction may be a good prognostic indicator for CRC, and, in some cases, may even surpass the TNM staging system, our study suggests that this may not be the case for all populations.

## 5. Conclusions

Overall, our data show that the presence of CD3^+^, CD8^+^, or FoxP3^+^T lymphocytes in the tumor invasive margin are associated with the pathogenesis of CRC. However, we did not find any association between lymphocyte infiltrations and the overall survival of CRC patients, although high FoxP3^+^ T lymphocyte infiltrations in the tumor invasive margins resulted in an increased overall survival of 14 months.

## Figures and Tables

**Table 1:** Demographic and baseline characteristics of the patients.

Parameter	N	Parameter	N	Parameter	N
Gender		Time to Diagnosis		TNM	
Male	120	<6 months	126	I-II	50
Female	64	>6 months	24	III-IV	134
Age (years)		Localization		Histological Type	
≤45	5	Colon	129	Adenocarcinoma	170
>45	179	Rectum	55	Mucinous	12
Previous tumors		CEA (ng/mL)		Signet ring and mucinous	2
Without	150	≤10	132	Differentiation	
With	34	>10	22	Well/Moderately	167
				Poorly/Undifferentiated	16
Family History		Metastasis		Lymph node metastasis	
Without	156	No	138	Without	106
With	15	Yes	46	With	77
Presentation		Tumor size (cm)			
Asymptomatic	34	≤4.5	107		
Symptomatic	150	>4.5	70		

**Table 2:** Distribution of cases according to the expression of CD3, FoxP3 and CD8 markers.

IHC Markers	CD3			FoxP3			CD8		
	N	Low (%)	High (%)	N	Low (%)	High (%)	N	Low (%)	High (%)
Intratumor Tissue	194	91 (47%)	103 (53%)	194	92 (47%)	102 (53%)	187	90 (48%)	97 (52%)
Tumor Margin Tissue	192	79 (41%)	113 (59%)	194	80 (41%)	114 (59%)	185	78 (42%)	107 (58%)
Normal Adjacent Tissue	143	84 (59%)	61 (43%)	146	84 (58%)	62 (42%)	139	82 (59%)	57 (41%)

**Table 3:** Correlation of marker’s expression according to the different regions analyzed.

Parameter	CD3 Intratumor				CD3 Tumor Margin			
	N	Low (%)	High (%)	*p*	N	Low (%)	High (%)	*p*
CD3 Tumor margin								
Low (%)	78	52 (66.7)	26 (33.3)	<0.001	-	-	-	-
High (%)	112	37 (33)	75 (67)		-	-	-	
CD3 Normal adjacent								
Low (%)	84	44 (52.4)	40 (47.6)	0.114	83	46 (55.4)	37 (44.6)	<0.001
High (%)	59	23 (39)	36 (61)		60	13 (21.7)	47 (78.3)	
**Parameter**	**FoxP3 Intratumor**				**FoxP3 Tumor Margin**			
	**N**	**Low (%)**	**High (%)**	***p***	**N**	**Low (%)**	**High (%)**	***p***
FoxP3 Tumor margin								
Low (%)	79	55 (69.6)	24 (30.4)	<0.001	-	-	-	-
High (%)	114	38 (33.3)	76 (63.7)		-	-	-	
FoxP3 Normal adjacent								
Low (%)	84	44 (52.4)	40 (47.6)	0.175	83	47 (56.6)	36 (43.4)	<0.001
High (%)	61	25 (41)	36 (59)		61	14 (23)	47 (77)	
**Parameter**	**CD8 Intratumor**				**CD8 Tumor Margin**			
	**N**	**Low (%)**	**High (%)**	***p***	**N**	**Low (%)**	**High (%)**	***p***
CD8 Tumor margin								
Low (%)	78	53 (28.8)	24 (31.2)	<0.001	-	-	-	-
High (%)	105	35 (33.3)	70 (66.7)		-	-	-	
CD8 Normal adjacent								
Low (%)	82	43 (52.4)	39 (47.6)	0.189	81	46 (56.8)	35 (43.2)	<0.001
High (%)	56	23 (41.1)	33 (58.9)		55	14 (25.5)	41 (74.5)	

**Table 4:** Association of intratumor infiltration of CD3^+^, FoxP3^+^, and CD8^+^ T lymphocytes with clinical data.

Parameter	CD3				FoxP3				CD8			
	N	Low (%)	High (%)	*p*	N	Low (%)	High (%)	*p*	N	Low (%)	High (%)	*p*
Gender												
Male	120	57 (47.5)	63 (52.5)	0.273	123	60 (48.8)	63 (51.2)	0.391	116	57 (49.1)	59 (50.9)	0.477
Female	64	25 (39.1)	39 (60.9)		64	27 (42.2)	37 (57.8)		62	27 (43.5)	35 (56.5)	
Age (years)												
≤45	5	3 (60)	2 (40)	0.481	5	3 (60)	2 (40)	0.540	5	3 (60)	2 (40)	0.561
>45	179	79 (44.1)	100 (55.9)		182	84 (46.2)	9 (53.8)		173	81 (46.8)	92 (53.2)	
Previous tumors												
Without	150	67 (44.7)	83 (55.3)	0.954	152	71 (46.7)	81 (53.3)	0.915	147	69 (46.9)	78 (53.1)	0.883
With	34	15 (44.1)	19 (55.9)		35	16 (45.7)	19 (54.3)		31	15 (48.4)	16 (51.6)	
Family History												
Without	156	73 (4.8)	83 (53.2)	0.135	159	77 (48.4)	82 (51.6)	0.106	152	74 (48.7)	78 (51.3)	0.214
With	15	4 (26.7)	11 (73.3)		15	4 (26.7)	11 (73.3)		13	4 (30.8)	9 (69.2)	
Presentation												
Asymptomatic	34	18 (52.9)	16 (47.1)	0.276	34	19 (55.9)	15 (44.1)	0.226	33	18 (54.5)	15 (45.5)	0.348
Symptomatic	150	64 (42.7)	86 (57.3)		153	68 (44.4)	85 (55.6)		145	66 (45.5)	79 (54.5)	
Time to Diagnosis												
<6 months	126	57 (45.2)	69 (54.8)	0.145	129	61 (47.3)	68 (52.7)	0.101	121	59 (48.8)	62 (51.5)	0.078
>6 months	24	7 (29.2)	17 (70.8)		24	7 (29.2)	17 (70.8)		24	7 (29.2)	17 (70.8)	
Localization												
Colon	129	57 (44.2)	72 (55.8)	0.874	131	61 (46.6)	70 (53.4)	0.986	124	60 (48.4)	64 (51.6)	0.628
Rectum	55	25 (45.5)	30 (54.5)		56	26 (46.4)	30 (53.6)		54	24 (44.4)	30 (55.6)	

**Table 5:** Association of intratumor infiltration of CD3^+^, FoxP3^+^, and CD8^+^ T lymphocytes with pathological data.

Parameter	CD3				FoxP3				CD8			
	N	Low (%)	High (%)	*p*	N	Low (%)	High (%)	*p*	N	Low (%)	High (%)	*p*
CEA (ng/mL)												
≤10	132	57 (43.2)	75 (56.8)	0.075	134	60 (44.8)	74 (55.2)	0.153	131	60 (45.8)	71 (54.2)	0.097
>10	22	14 (63.6)	8 (36.4)		23	14 (60.9)	9 (30.1)		18	12 (66.7)	6 (33.3)	
Metastasis												
No	138	57 (41.3)	81 (58.7)	0.123	141	61 (43.3)	80 (56.7)	0.117	135	60 (44.4)	76 (55.6)	0.193
Yes	46	25 (54.3)	21 (45.7)		46	26 (56.5)	20 (43.5)		43	24 (55.8)	19 (44.2)	
Tumor Size (cm)												
≤4.5	107	49 (45.8)	58 (54.2)	0.844	110	52 (47.3)	58 (52.7)	0.942	103	49 (47.6)	54 (52.4)	0.830
>4.5	70	31 (44.3)	39 (55.7)		69	33 (47.8)	36 (52.2)		67	33 (49.3)	34 (50.7)	
Histological Type												
Adenocarcinoma	170	75 (44.1)	95 (55.9)	0.913	172	79 (45.9)	93 (54.1)	0.751	163	76 (46.6)	87 (53.4)	0.773
Mucinous	12	6 (50)	6 (50)		12	6 (50)	6 (50)		12	6 (50)	6 (50)	
Signet ring and mucinous	2	1 (50)	1 (50)		3	2 (66.7)	1 (33.3)		3	2 (66.7)	1 (33.3)	
Differentiation												
Well/Moderately	167	73 (43.7)	94 (56.3)	0.473	168	77 (45.8)	91 (54.2)	0.425	169	74 (46.5)	85 (53.4)	0.458
Poorly/Undifferentiated	15	8 (53.3)	7 (46.7)		16	9 (56.3)	7 (43.8)		16	9 (56.3)	7 (43.8)	
Lymph Node Metastasis												
Without	106	44 (41.5)	62 (58.5)	0.292	106	45 (42.5)	61 (57.5)	0.126	101	44 (43.6)	57 (56.4)	0.17
With	77	38 (49.4)	39 (50.6)		78	42 (53.8)	36 (46.2)		74	40 (54.1)	34 (45.9)	
TNM												
I-II	50	21 (42)	29 (58)	0.669	51	20 (39.2)	31 (60.8)		48	19 (39.5)	29 (60.4)	0.201
III-IV	134	61 (45.5)	73 (54.5)		135	67 (49.6)	68 (50.4)		129	65 (50.4)	64 (49.6)	
Overall survival												
Months ± SD	184	106.8 ± 7.5	93.3 ± 7.2	0.294	187	103.4 ± 7.2	98.1 ± 7.3	0.598	178	103.3 ± 7.5	94.0 ± 7.5	0.358

**Table 6:** Association of tumor margin infiltration of CD3^+^, FoxP3^+^, and CD8^+^ T lymphocytes with clinical data.

Parameter	CD3				FoxP3				CD8			
	N	Low (%)	High (%)	*p*	N	Low (%)	High (%)	*p*	N	Low (%)	High (%)	*p*
Gender												
Male	118	48 (40.7)	70 (59.3)	0.303	122	51 (41.8)	71 (58.2)	0.296	115	49 (42.6)	66 (57.4)	0.529
Female	62	22 (35.5)	40 (64.5)		63	23 (36.5)	40 (63.5)		61	23 (37.5)	38 (62.3)	
Age (years)												
≤45	5	2 (40)	3 (60)	0.646	5	2 (40)	3 (60)	0.684	5	2 (40)	3 (60)	0.967
>45	175	68 (38.9)	108 (61.1)		180	72 (40)	108 (60)		171	70 (40.9)	101 (59.1)	
Previous tumors												
Without	148	58 (39.2)	90 (60.8)	0.513	151	61 (40.4)	90 (59.6)	0.488	146	59 (40.4)	87 (56.9)	0.767
With	32	12 (37.5)	20 (62.5)		34	13 (38.2)	21 (61.8)		30	13 (43.3)	17 (56.7)	
Family History												
Without	152	57 (37.5)	95 (62.5)	0.407	156	60 (38.5)	96 (61.5)	0.437	149	58 (38.9)	91 (61.1)	0.418
With	16	7 (43.8)	8 (56.6)		16	7 (43.8)	9 (56.3)		14	7 (50)	7 (50)	
Presentation												
Asymptomatic	32	10 (31.3)	22 (68.8)	0.22	33	11 (33.3)	22 (66.7)	0.254	32	10 (31.3)	22 (68.8)	0.219
Symptomatic	148	60 (40.5)	88 (59.5)		152	63 (41.4)	89 (58.6)		144	62 (43.1)	82 (56.9)	
Time to Diagnosis												
<6 months	125	52 (41.6)	73 (58.4)	0.325	128	54 (42.2)	74 (57.8)	0.424	120	53 (44.2)	67 (55.8)	0.547
>6 months	23	8 (34.8)	15 (65.2)		24	9 (37.5)	15 (62.5)		24	9 (37.5)	15 (62.5)	
Localization												
Colon	126	51 (40.5)	75 (59.5)	0.310	131	55 (42)	76 (58)	0.245	124	55 (44.4)	69 (55.6)	0.151
Rectum	54	19 (35.2)	35 (64.8)		54	19 (35.2)	35 (64.8)		52	17 (32.7)	35 (67.3)	

**Table 7:** Association of tumor margin infiltration of CD3^+^, FoxP3^+^, and CD8^+^ T lymphocytes with pathological data.

Parameter	CD3				FoxP3				CD8			
	N	Low (%)	High (%)	*p*	N	Low (%)	High (%)	*p*	N	Low (%)	High (%)	*p*
CEA (ng/mL)												
≤10	128	44 (34.4)	84 (65.6)	0.026	132	48 (36.4)	84 (63.8)	0.057	129	48 (37.2)	81 (62.8)	0.053
>10	22	13 (59.1)	9 (40.9)		23	13 (56.5)	10 (43.5)		18	11 (61.1)	7 (38.9)	
Metastasis												
No	134	47 (35.1)	87 (64.9)	0.054	139	51 (36.7)	88 (63.8)	0.078	133	50 (37.6)	83 (62.4)	0.116
Yes	46	23 (50)	23 (50)		46	23 (50)	23 (50)		43	22 (51.2)	21 (48.8)	
Tumor Size (cm)												
≤4.5	103	42 (40.8)	61 (59.2)	0.185	107	46 (43.0)	61 (57)	0.116	56	44 (44)	56 (56)	0.186
>4.5	70	23 (32.9)	47 (67.1)		70	23 (32.9)	47 (67.1)		45	23 (33.8)	45 (66.2)	
Histological Type												
Adenocarcinoma	166	62 (37.3)	104 (62.7)	0.336	170	65 (38.2)	105 (61.8)	0.248	161	63 (39.1)	98 (60.9)	0.281
Mucinous	12	7 (58.3)	5 (41.7)		12	7 (58.3)	5 (41.7)		12	7 (58.3)	5 (41.7)	
Signet ring and mucinous	2	1 (50)	1 (50)		3	2 (66.7)	1 (33.3)		3	2 (66.7)	1 (33.3)	
Differentiation												
Well/Moderately	163	62 (38)	101 (62)	0.347	166	65 (39.2)	101 (60.8)	0.279	157	63 (40.1)	94 (59.9)	0.444
Poorly/undifferentiated	15	7 (46.7)	8 (53.3)		16	8 (50)	8 (50)		16	8 (50)	8 (50)	
Lymph Node Metastasis												
Without	103	32 (31.1)	71 (68.9)	0.010	105	33 (31.4)	72 (68.6)	0.004	100	32 (32)	68 (68)	0.003
With	76	38 (50)	38 (50)		78	41 (52.6)	37 (47.4)		74	40 (54.1)	34 (45.9)	
TNM												
I-II	47	12 (25.5)	35 (74.5)	0.021	48	12 (25)	36 (75)	0.012	45	12 (26.7)	33 (73.3)	0.022
III-IV	133	58 (43.6)	76 (56.4)		136	62 (45.6)	74 (54.4)		130	60 (46.2)	70 (53.8)	
Overall survival												
Months ± SD	180	93.0 ± 8.5	106.4 ± 6.7	0.117	185	92.8 ± 8.1	106.8 ± 6.7	0.092	176	93.2 ± 8.3	100.2 ± 7.0	0.224

## Data Availability

The data presented in this study are available upon request to the corresponding author.
